# Experimental Study on the Strength and Durability of Manufactured Sand HPC in the Dalian Bay Undersea Immersed Tube Tunnel and Its Engineering Application

**DOI:** 10.3390/ma17205003

**Published:** 2024-10-12

**Authors:** Yuefeng Sun, Shanshan Song, Hongfa Yu, Haiyan Ma, Yu Xu, Guojia Zu, Yang Ruan

**Affiliations:** 1 CCCC First Harbor Bureau No. 3 Engineering Co., Ltd., Dalian 116000, China; 13079807988@126.com (Y.S.); ruanyang0312@126.com (G.Z.); 15141165201@163.com (Y.R.); 2 Department of Civil and Airport Engineering, Nanjing University of Aeronautics and Astronautics, Nanjing 210016, China; mahaiyan@nuaa.edu.cn (H.M.); 15951975860@163.com (Y.X.); 3 School of Civil Engineering, Sanjiang University, Nanjing 210012, China

**Keywords:** manufactured sand, high-performance concrete, stone powder content, mechanical properties, durability, Dalian bay undersea immersed tube tunnel

## Abstract

The usage of manufactured sand concrete is widespread in modern engineering, and it is important to study its performance to improve the overall engineering quality. This paper presents an experimental study on the working performance and durability of 12 groups of manufactured sand high-performance concrete (MSHPC) with varying mix ratios, in the context of the construction of the Dalian Bay undersea immersed tube tunnel. The study reveals that the stone powder content significantly affects the physical and mechanical properties, as well as the durability, of manufactured sand concrete. At an approximately 9% stone powder content, the concrete achieves the highest slump and best workability. However, excessive stone powder reduces early crack resistance. Furthermore, an optimal stone powder content (ranging from 5% to 13%) enhances the compressive strength, with the 28-day compressive strength reaching 60 MPa at a 13% stone powder content, while the effect on the splitting tensile strength is negligible. The stone powder content does not significantly impact impermeability and frost resistance, but at 7–9%, the RCM method shows the lowest chloride ion diffusion coefficient. Additionally, a lower water–binder ratio enhances resistance to chloride ion diffusion. High-performance RCM concrete with a 9% stone powder content was used in the construction of the Dalian Bay Cross-Harbor Tunnel, achieving 28-day and 56-day compressive strengths of C45 and C50, respectively, an impermeability grade of P14, a chloride ion diffusion coefficient of 1.9 × 10^−12^ m^2^/s, and a frost durability index of 92%, meeting the project’s 100-year lifespan design requirements.

## 1. Introduction

Due to the rapid growth of the construction industry and increased environmental regulations, natural sand, primarily river sand, is not sufficient to meet the demands of construction. In 2002, the state enforced the “Regulations on the Management of River Sand Mining”, worsening the shortage of construction sand. Consequently, manufactured sand has emerged as a substitute for natural sand in concrete production. Different countries and regions have established specific grading standards for manufactured sand, such as ASTM C33-2008 [1] in the United States, JIS A 5005-2009 [2] in Japan, AS 2758.1-2014 [3] in Australia, and GB/T 14684-2011 [4] in China. Many researchers have studied the workability, mechanical properties, and durability of concrete containing manufactured sand [5,6,7]. The content of stone powder in manufactured sand significantly impacts the performance of this concrete. For instance, Bonavetti et al. [8] reported that increasing the limestone powder content enhances the early compressive strength of the mortar; Tang Yu et al. [9] observed that the flexural and compressive strengths of concrete made with manufactured sand are superior to those of concrete made with standard sand at an equivalent stone powder content; Chen Fengbin et al. [10] determined that the optimal stone powder content for C20 concrete ranges from 10% to 15%; Liu Xiumei et al. [11] concluded that for C40 concrete, the optimal range is 7% to 11%; and Jiang Zhengwu et al. [12] identified that the optimal stone powder content for high-strength concrete is 5% to 7%. Manufactured sand has been widely used in China for many years, notably in significant engineering projects such as the South-to-North Water Diversion Project [13], the Sichuan–Tibet Railway [14], and the Pingtang Special Bridge [15]. However, there is still insufficient research on the application of high-performance concrete (HPC) made with manufactured sand, particularly concerning its durability in marine environments in northern China.

This paper investigates the effect of different stone powder contents on the strength and durability of HPC made with manufactured sand for the construction of a submarine immersed tube tunnel in Dalian Bay. The mechanism sand used in this study is made of limestone, where stone powder is the part of the mechanism sand with a very low particle density. The production of limestone is a relatively straightforward process, with reliable equipment operation, low production costs, and favorable economic benefits. Consequently, limestone is the preferred rock type for the production of mechanism sand. Wang et al. reported that limestone powder can influence the properties of cement-based materials via filler, nucleation, dilution and chemical effects [16]. Liao et al. found that adding an appropriate amount of limestone stone powder can promote the hydration of cement, and can improve the compressive strength of concrete [17]. An optimized HPC mix design for manufactured sand is proposed based on these findings. Additionally, concrete mix ratios for service lives of 100 and 120 years were established during the project preparation stage [18], and studies on the durability and lifespan design of concrete structures in different marine exposure zones were conducted [19]. In the actual construction process, HPC was prepared using manufactured sand, and the optimal stone powder content for HPC was determined through testing. This optimized mix was successfully applied in the construction of the Dalian Bay submarine immersed tube tunnel. The results of this study not only provide theoretical support and technical guidance for the development of HPC technology using manufactured sand in China, but also provide a valuable reference for the promotion of this technology to meet the design requirements of major marine concrete structures with a service life of 100 to 120 years.

## 2. Raw Material and Mix Ratio

### 2.1. Raw Materials

Cement: P·O 42.5 silicate cement from Dalian Cement Plant was used. Its basic physical properties are presented in Table 1, and its chemical composition is detailed in Table 2.

Fly Ash (FA): Class I fly ash from Dalian Huaneng was utilized, with a water content of 0.1%, a water requirement ratio of 91%, a fineness (0.045 mm) of 4.6%, and an SO_3_ content of 1%. The chemical composition is shown in Table 2.

Slag (SG): S95-grade ground slag from Dalian Jinqiao Company (Dalian, China) was used, with a density of 2.88 g/cm^3^, a specific surface area of 415 m^2^/kg, a mobility ratio of 101%, a water content of 0.1%, and a chloride ion content of 0.02%. The main chemical composition is detailed in Table 2.

**Figure 1:**
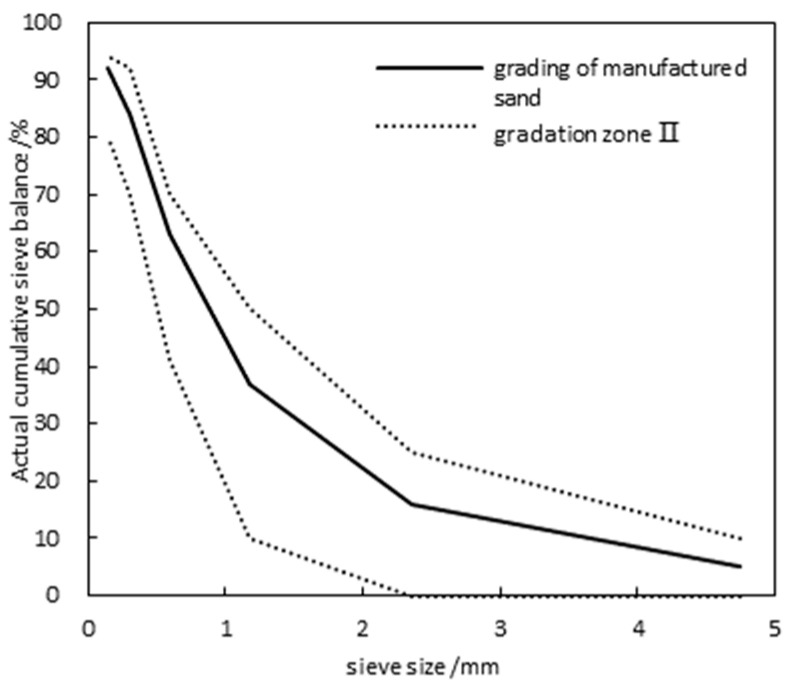
Manufactured sand grading curve.

Manufactured Sand: Limestone was selected as the source rock for the manufactured sand. The MB value of the sand does not exceed 1.4, and it falls within grading area II, with a fineness modulus of 2.8, an apparent density of 1520 kg/m^3^, a mud content of no more than 0.5%, and a water absorption rate not exceeding 2.0%. The content of hazardous substances complies with the Type I limits for manufactured sand. The grading curve is shown in Figure 1.

**Figure 2:**
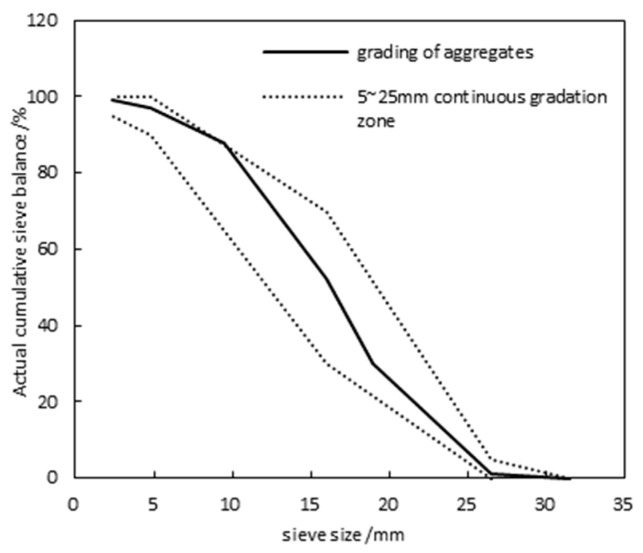
Aggregates grading curve.

Stone: Crushed limestone from Dalian was used, with an apparent density of 2720 kg/m^3^, a bulk density of 1420 kg/m^3^, a maximum particle size of 25 mm, a mud content of 0.8%, and a needle flake particle content of 5%. The crushing index is 9%, and the material follows a continuous grading from 5 to 25 mm. The grading curve is shown in Figure 2.

**Figure 3:**
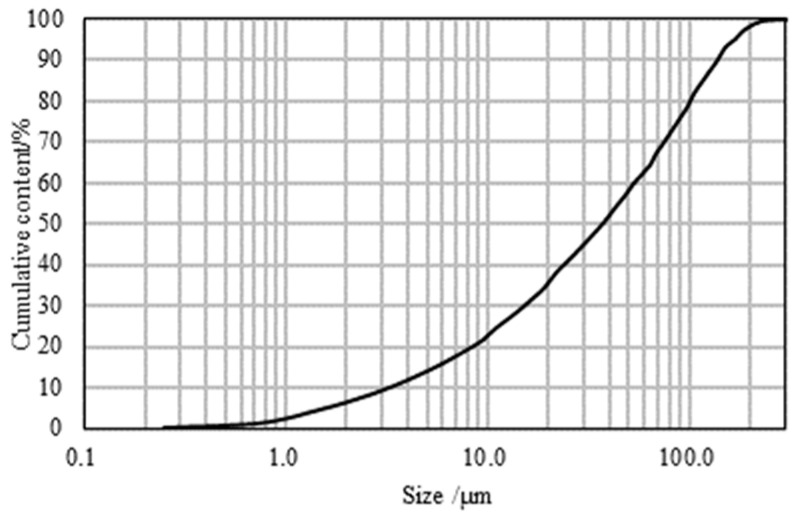
Stone powder grading curve.

Stone Powder: Limestone was used as the raw material, with particles smaller than 0.08 mm accounting for 71.25% of the content. The median particle size (D50) is 38.94 μm. The grading curve is shown in Figure 3. The stone powder content in the manufactured sand was controlled by back-mixing.

Water-Reducing Admixture: A SW-A polycarboxylic acid-based high-performance water-reducing agent produced by Dalian Shenwei Building Material Products Co. (Dalian, China) was used. The chemical composition includes K_2_O at 1.02%. The recommended dosage is 1.0% of the cementitious material, with a water reduction rate of 26.3%.

Air-Entraining Agent: A PC-2 rosin thermopolymer air-entraining agent from Qingdao Keli Building Materials Co., Ltd. (Qingdao, China) was used. The main component is sodium rosinate (C_19_H_29_COONa). The agent is a liquid with a density of 1.01 g/cm^3^, a solid content of 3.7%, a pH value of 12.2, no chloride content, and an equivalent alkali content (Na_2_O) of 0.68%. The foam degree (hand shaking ≥ 40) is 45%, and the recommended dosage is 0.012%, with a water reduction rate of 7.5%.

Water: Tap water from Dalian city was used.

### 2.2. Mix Ratio

Three series of HPC mixes with manufactured sand were designed to investigate the effects of different factors on the workability and durability of HPC. The first series (JA5-1, JA7-1, JA9-1, JA11-1, and JA13-1) had a water–binder ratio of 0.32, with 15% fly ash, 15% slag, and a stone powder content ranging from 5% to 13%. The second series (JA5-2, JA7-2, JA9-2, JA11-2, and JA13-2) also had a water–binder ratio of 0.32, with 15% fly ash, 20% slag, and a stone powder content between 5% and 13%. The third series (JA9-1, JB9-1, and JC9-1) explored the effects of different water–binder ratios (0.32, 0.34, and 0.36), with a constant 15% fly ash, 15% slag, and 9% stone powder content. The specific mix proportions for all HPC specimens made with manufactured sand are detailed in Table 3.

## 3. Test Method

### 3.1. Preparation and Curing of Concrete Specimens

The cement, stone, sand, mineral admixtures, and additives were first dry mixed in a mixer for 1 min. Water was then added, and mixing continued for an additional 3 min. After the mixture was discharged, the slump was measured before casting and vibrating the concrete into molds. The specimens were categorized into three sizes: 100 mm × 100 mm × 400 mm prisms (60 groups, 180 pieces), 150 mm × 150 mm × 150 mm cubes (120 groups, 360 pieces), and Ø150 mm × 150mm cylinders (120 groups, 360 pieces). The specimens were kept in their molds for 24 h, after which the molds were removed, and the specimens were placed in a curing room with controlled conditions of 20 ± 2 °C and a humidity greater than 95%.

### 3.2. Early Crack Resistance Test

Concrete is susceptible to cracking during early hydration, which may have a direct impact on its performance and service life. Therefore, it is essential to evaluate the early cracking resistance of concrete. This test evaluates the concrete’s resistance to induced cracking under dry and ventilated conditions from the time it is cast to the early hardening stage. The testing was conducted according to the JC/T2234-2014 specification, “Test Method for Early Cracking Resistance of Cement” [20].

### 3.3. Strength Test

The specimens for the concrete compressive strength and split tensile strength tests (referred to as split tensile strength) were 150 mm × 150 mm × 150 mm in size. Testing was conducted in accordance with the GB/T 50081-2019 standard, “Test Methods for Physical and Mechanical Properties of Concrete” [21].

### 3.4. Test of Freezing Resistance and Salt Freezing Resistance

In China’s northern region, which experiences cold winters, addressing freeze–thaw damage in marine concrete is essential. The evaluation of freeze–thaw resistance was conducted using two methods from the GB/T 50082-2009 standard, “Long-Term Performance and Durability Test Methods for Ordinary Concrete” [22]: the rapid freeze–thaw test and the one-sided freeze–thaw test.

**Figure 4:**
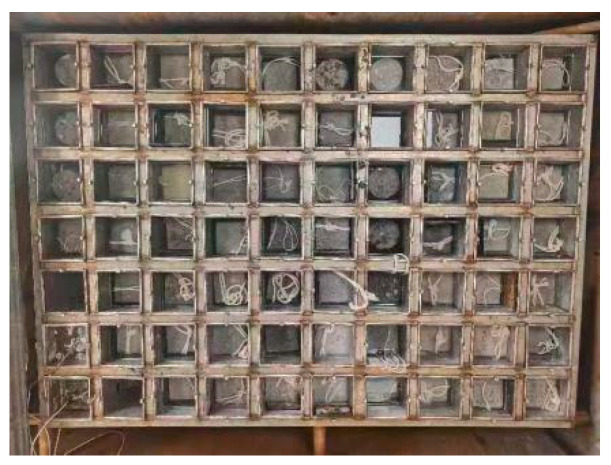
Rapid freeze–thawing experiment of concrete specimen.

For the rapid freeze–thaw test, 100 mm × 100 mm × 400 mm prismatic specimens were immersed in Dalian Bay seawater, with the seawater composition detailed in Table 4. The test utilized the KDR-28V model experimental equipment from Beijing Sanshihang Measurement and Control Technology Co., Ltd. (Beijing, China), as shown in Figure 4. The dynamic elastic modulus and mass change were measured every 25 cycles, with a total of 600 cycles performed. The freeze–thaw resistance was evaluated using the relative dynamic elastic modulus, mass loss rate, and freeze durability index, as defined by the following:

*K*_n_ = *P* × *N*/300 (1)

where *P* is the relative modulus of elasticity after *N* freeze–thaw cycles, and *N* is the number of cycles until the relative dynamic modulus of elasticity drops below 60% or the mass loss reaches 5%.

For the one-sided freeze–thaw test, cubic specimens measuring 150 mm × 150 mm × 150 mm were cut into 150 mm × 150 mm × 75 mm test pieces. The Wuzhou Refrigeration LSBLG130Z11 model experimental equipment was used. The specimens were exposed to a 3% NaCl solution for freezing and thawing, with the freezing and thawing surface area measuring 150 mm × 150 mm. Epoxy resin was applied to the sides of the specimens and air-dried before saturating the specimens with water every two days for a total of three saturations. In this process, the bottom surface of the specimen was immersed in salt solution, and the side of the specimen was not affected, with only the bottom surface being subjected to freezing and thawing damage; this simulates the freezing and thawing process of road pavement. The mass of the specimens after each saturation was recorded. Afterwards, 28 freeze–thaw cycles were conducted, with the mass loss measured every four cycles. The frost resistance was assessed based on the total mass loss per unit area or the relative dynamic modulus of elasticity.

### 3.5. Impermeability Test

The water penetration height method, as specified in the GB/T 50082-2009 standard, “Test Method for Long-Term Performance and Durability of Ordinary Concrete,” was employed to evaluate concrete impermeability. The specimens were cylindrical with dimensions of Ø175 mm × 185 mm × 150 mm. After standard curing for 28 days, the specimens were subjected to the impermeability apparatus, where the water pressure was maintained at (1.2 ± 0.05) MPa for 24 h. The pressurization process was completed within 5 min, and the time taken for the pressure to stabilize was recorded with a precision of 1 min.

After 24 h, the specimens were split, and the water seepage height was measured using a steel ruler. Measurements were taken along the seepage contour line at equal intervals of 10 points on the specimen’s surface, with readings accurate to 1 mm. The average seepage height of the specimen was calculated and rounded to the nearest 1 mm. The average water penetration height for each group of 6 specimens was determined by averaging the water penetration heights of the individual specimens.

### 3.6. Electrical Flux Permeability and RCM Method for Unsteady-State Electromigration Chloride Diffusion Test

To conduct the electrical flux permeability and RCM tests, a 25 mm section was removed from the casting surface of the 150 mm cube concrete specimens using a cutting machine. The cuts were made to avoid the possible presence of floating and mortar layers on the surface of the concrete casting and to ensure that the measured concrete data were representative. Subsequently, cylindrical specimens that were 100 mm in diameter and 50 mm in height were prepared using a drilling machine. These specimens were then used for the electrical flux and RCM tests. The testing procedures followed the guidelines outlined in the GB/T 50082-2009 standard, “Long-Term Performance and Durability Test Methods for Ordinary Concrete” [22].

## 4. Results and Discussion

### 4.1. Workability of MSHPC

#### 4.1.1. Effect of Stone Powder Content on the Slump of MSHPC

**Figure 5:**
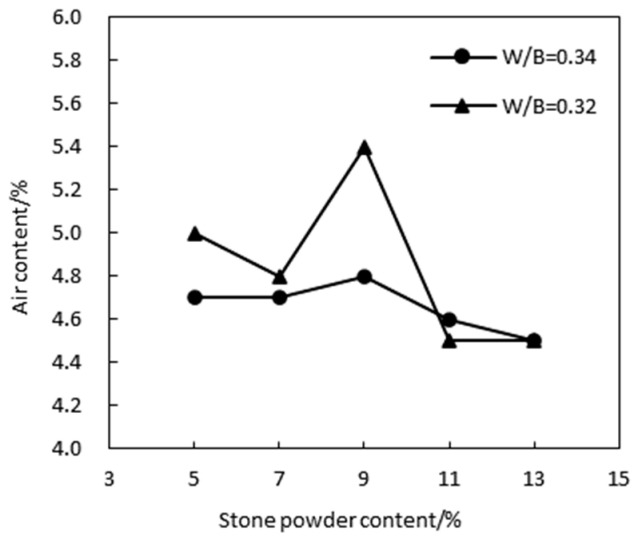
Effect of the stone powder content on slump.

The workability of concrete is an important parameter for assessing the performance of mass concrete. The stone powder content in manufactured sand influences the slump of HPC. Figure 5 illustrates how the HPC slump varies with the stone powder content. The data show that the slump of HPC using manufactured sand consistently remains above 200 mm. Initially, as the stone powder content increases, the slump increases but eventually begins to decrease. The maximum slump and optimal workability are observed at an approximately 9% stone powder content. Beyond this point, the slump decreases significantly. This reduction is attributed to the angular and rough surface of manufactured sand particles, which increases the friction between aggregates and thereby reduces slump. Additionally, the larger surface area of the stone powder enhances the cohesion of the concrete mix.

#### 4.1.2. Effect of Stone Powder Content on Air Content of HPC with Manufactured Sand

**Figure 6:**
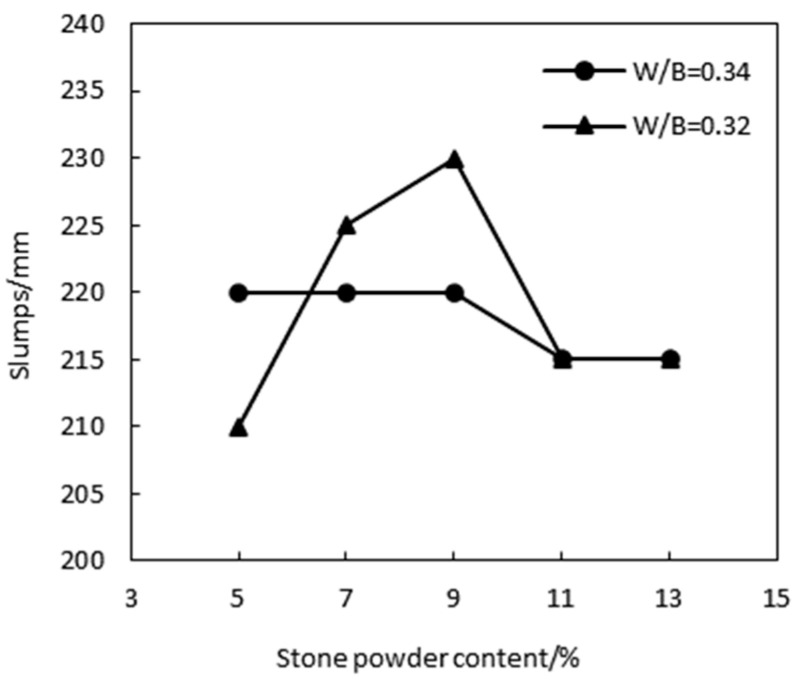
Effect of the stone powder content on the air content of MSHPC.

Figure 6 illustrates the air content of HPC with manufactured sand at water–binder ratios of 0.32 and 0.34 across various stone powder contents. The results indicate that the air content reaches its maximum value (4.8–5.4%) at a 9% stone powder content, aligning with the optimal stone powder content determined from the slump test.

### 4.2. Early Cracking Resistance of MSHPC

Table 5 presents the experimental results for the early cracking resistance of HPC flat slabs using manufactured sand, with a water–binder ratio of 0.32, fly ash content of 15%, and slag content of 15%. The data indicate that the total cracked area per unit of manufactured sand HPC increases progressively with the stone powder content. Specifically, when the stone powder content rises from 5% to 13%, the total cracked area per unit increases by 78.2%. This suggests that a higher stone powder content diminishes the early cracking resistance of HPC with manufactured sand.

### 4.3. Compressive Strength and Splitting Tensile Strength of MSHPC

#### 4.3.1. Effect of Stone Powder Content on MSHPC Strength

**Figure 7:**
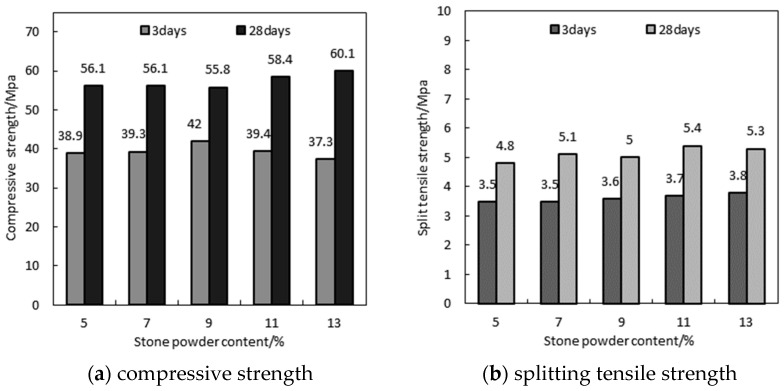
Compressive strength and split tensile strength of MSHPC with different stone powder contents (W/B = 0.32).

The crack resistance in large-volume immersed pipe concrete is a crucial challenge in construction. The splitting tensile strength is an important indicator of temperature-controlled crack resistance. Figure 7 illustrates the compressive and splitting tensile strengths of MSHPC (with a water–binder ratio of 0.32) at 3 and 28 days, with varying stone powder contents in the manufactured sand. The data reveal that the stone powder content has a significant impact on the compressive strength of MSHPC, but its effect on the splitting tensile strength is minimal. At 3 days of curing, the compressive strength initially increases and then decreases with a higher stone powder content, peaking at 42 MPa with a 9% stone powder content. At 28 days, due to more complete hydration, MSHPC achieves a C50 strength grade, with the highest compressive strength observed at a 13% stone powder content. It is also essential to undertake the reasonable selection and arrangement of steel reinforcement within the concrete during the engineering and construction process. This is in order to reduce the number of cracks and the width of cracks produced when the concrete member is under pressure [23].

The following points explain how stone powder enhances the compressive strength of MSHPC: (1) Filling Effect: The stone powder in mechanism sand mainly plays the role of physical filling in concrete, which can have a good micro-aggregate filling effect and improve the mechanical properties of concrete [24]. (2) Microstructure Optimization: During cement hydration, stone powder participates in reactions and forms monocarboaluminate crystals, enhancing the microstructure of HPC. This improves the overall structural compactness. Stone powder also improves the interfacial transition zone between the cement paste and aggregate, increasing the bond strength at the interface [25,26]. (3) Water Reduction: Stone powder acts as a lubricant, enhancing the fluidity and workability of the HPC. This results in a more homogeneous and dense concrete mixture during pouring and vibration. (4) Diminished Effect Beyond Optimum Content: When the stone powder content exceeds the optimal level, the cement reaches its maximum hydration limit. The additional stone powder does not contribute to new hydration products, and replaces some of the cement. This leads to a reduction in hydrated calcium silicate gel and a consequent decrease in compressive strength.

#### 4.3.2. Effect of Water–Binder Ratio on Strength of MSHPC

**Figure 8:**
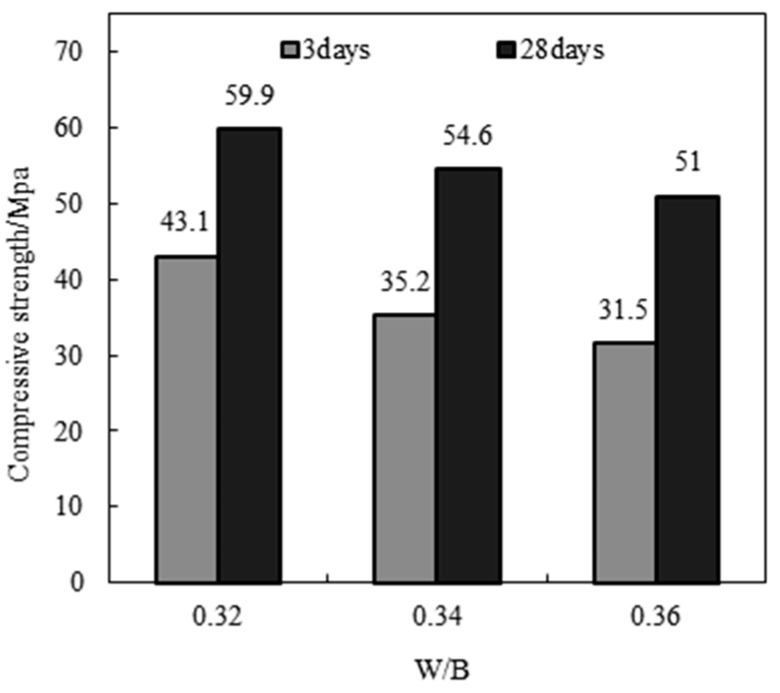
Effect of the water cement ratio on the compressive strength of MSHPC (stone powder 9%).

Figure 8 illustrates the influence of the water–binder ratio on the 3-day and 28-day compressive and splitting tensile strengths of MSHPC with a 9% stone powder content. The water–binder ratios considered were 0.32, 0.34, and 0.36. The results indicate that a lower water–binder ratio increases the compressive and splitting tensile strengths. For instance, comparing MSHPC samples JA9-1 and JA9-2, which both contain 9% stone powder, the 28-day compressive strength of the sample with 15% fly ash and 15% ground slag is 59.9 MPa, which is 6.3% higher than that of the sample with 15% fly ash and 20% ground slag.

### 4.4. Permeability of HPC with Manufactured Sand

**Figure 9:**
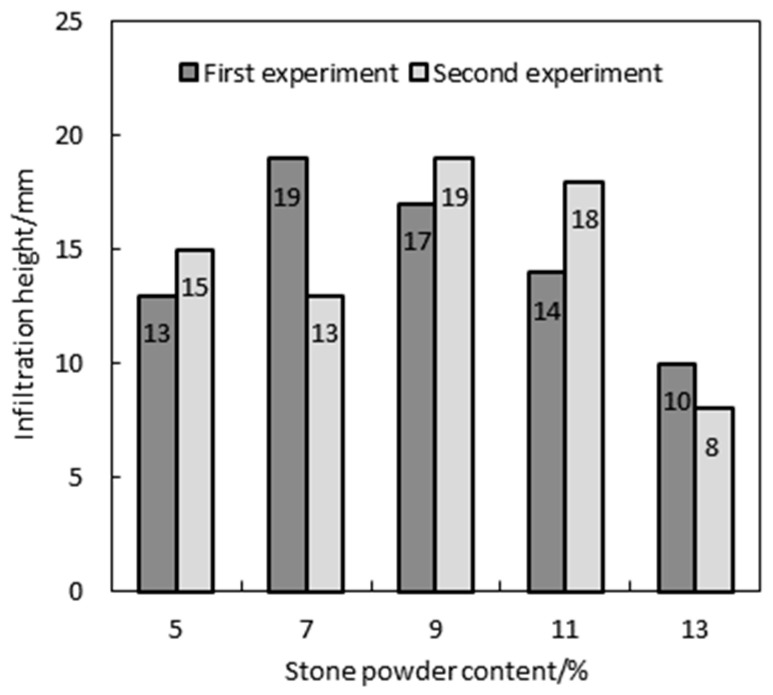
Effect of the stone powder content on MSHPC impermeability.

Figure 9 presents the permeability performance of HPC with manufactured sand at varying stone powder contents. Under a sustained water pressure of 1.2 MPa for 24 h, the maximum and minimum water seepage heights for HPC with stone powder contents ranging from 5% to 13% were 19 mm and 8 mm, respectively, with all samples exceeding a P14 permeability rating. The seepage height initially increases and then decreases with a rising stone powder content, showing a significant reduction in permeability and the strongest resistance when the stone powder content reaches 13%. This is because mixing an appropriate amount of stone powder into MSHPC can slow down exothermic hydration and improve the pore structure, thus improving the impermeability of concrete [27].

### 4.5. Frost Resistance and Salt Freezing Resistance of MSHPC

#### Frost Resistance of MSHPC in Seawater

**Figure 10:**
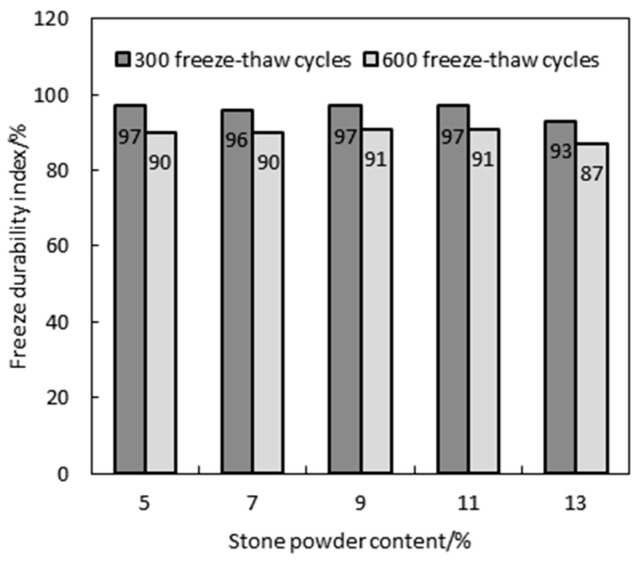
Effect of the stone powder content on the freezing resistance of MSHPC.

**Figure 11:**
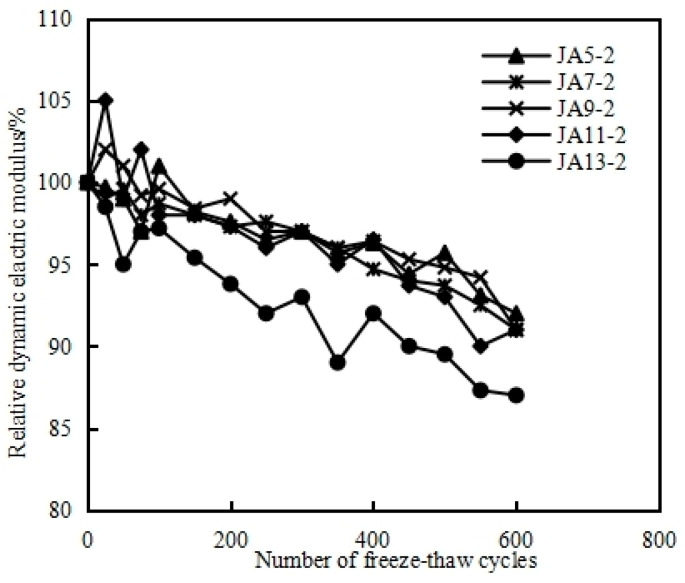
Relative elastic modulus of MSHPC under a freeze–thaw cycle.

The concrete structure of the submarine immersed tube tunnel in Dalian Bay must withstand the tidal zone and wave splash zone of the ocean, as well as the potential damage from freezing and thawing caused by seawater. Figure 10 shows the freeze durability indices of MSHPC with different stone powder contents after rapid freeze–thaw tests in Dalian seawater. Specifically, the water–binder ratio of the MSHPC specimens was 0.32, with a 15% fly ash dosing and 20% slag dosing, and the stone powder content ranged from 5% to 13%. As shown in the figure, the frost durability index of MSHPC specimens ranged from 87% to 97%, and all exceeded the durability index of 0.8. Figure 11 reflects the change in the relative dynamic elastic modulus of MSHPC specimens after 600 freeze–thaw cycles, where the relative dynamic elastic modulus did not fall below 60%, meeting the standard for 600 freeze–thaw cycles. The test results indicate that within 600 freeze–thaw cycles, there is no significant difference in the frost resistance test for concrete specimens with different stone powder contents, and all exhibited superior frost resistance in seawater.

At the entrances and exits of the immersed tube tunnels in Dalian Bay, sodium chloride is typically applied as a de-icing agent during winter to melt snow and ice. To evaluate the salt freezing resistance of MSHPC(HPC) under these conditions, a one-sided salt freezing test was conducted. The test specimens used had the same mix proportions as those in the frost resistance test. After 28 cycles of one-sided salt freezing, the specimens exhibited minimal mass loss and no significant degradation in the relative dynamic elastic modulus. These results indicate that MSHPC demonstrates excellent salt freezing resistance across various stone powder content conditions.

### 4.6. Electrical Flux of MSHPC

**Figure 12:**
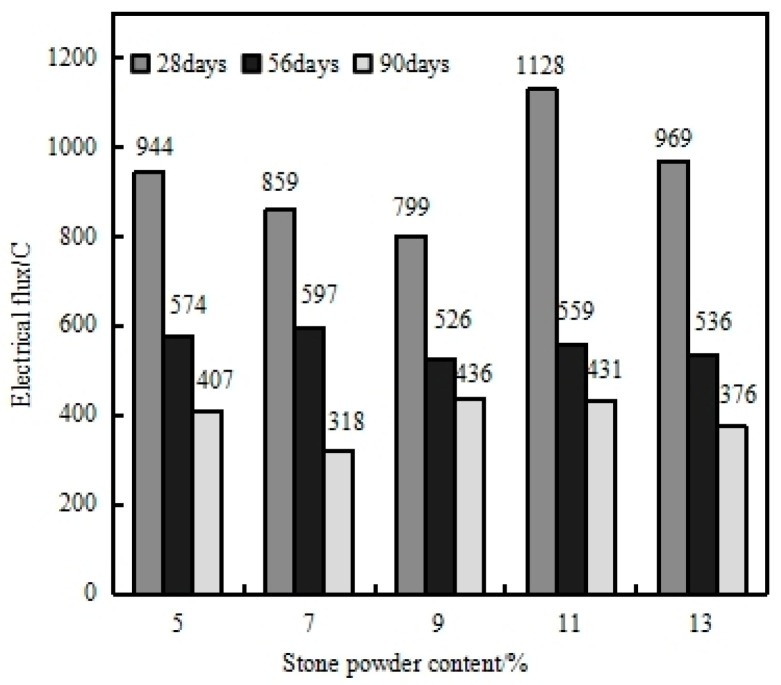
Effect of the stone powder content on the MSHPC electrical flux.

The chloride ion penetration resistance of MSHPC was evaluated through electrical flux tests at 28, 56, and 90 days on five different HPC mixtures: JA5-2, JA7-2, JA9-2, JA11-2, and JA13-2. The experimental results are presented in Figure 12, indicating that by day 56, the electrical flux of the specimens was below 1000C, meeting the requirements for HPC in harbor engineering.

**Figure 13:**
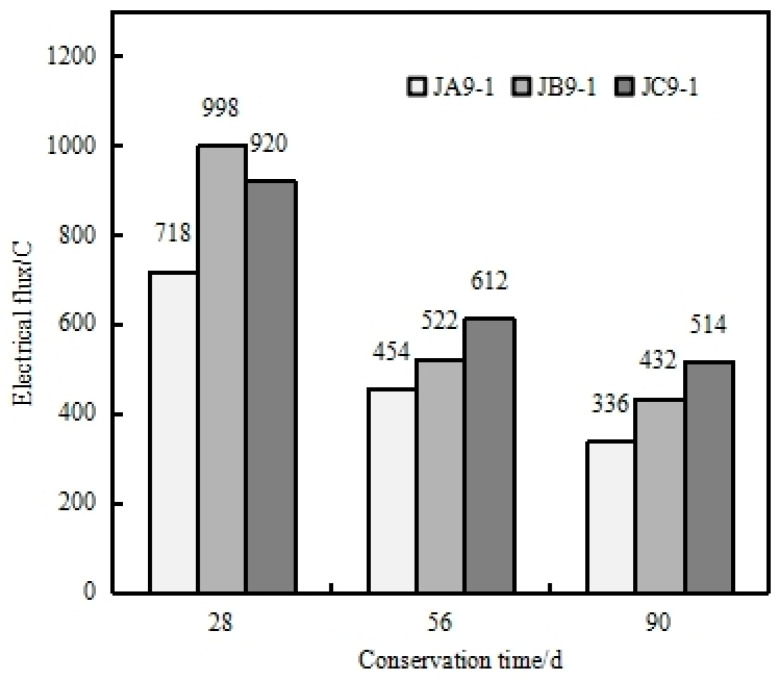
Electrical flux of MSHPC with different water adhesive ratios.

Figure 13 illustrates the electrical flux of three different HPC specimens—JA9-1, JB9-1, and JC9-1—at various ages, with water–binder ratios of 0.32, 0.34, and 0.36, respectively. The results show that as the water–binder ratio decreases, the electrical flux of the MSHPC decreases, leading to improved resistance to chloride ion penetration. Even at a higher water–binder ratio of 0.36 (JC9-1), the electrical flux at 56 days was 612C, meeting the requirements for chloride ion penetration resistance in marine engineering HPC.

### 4.7. Chloride Diffusion Resistance of MSHPC

#### 4.7.1. Effect of Stone Powder Content on Chloride Diffusion Coefficient of MSHPC

**Figure 14:**
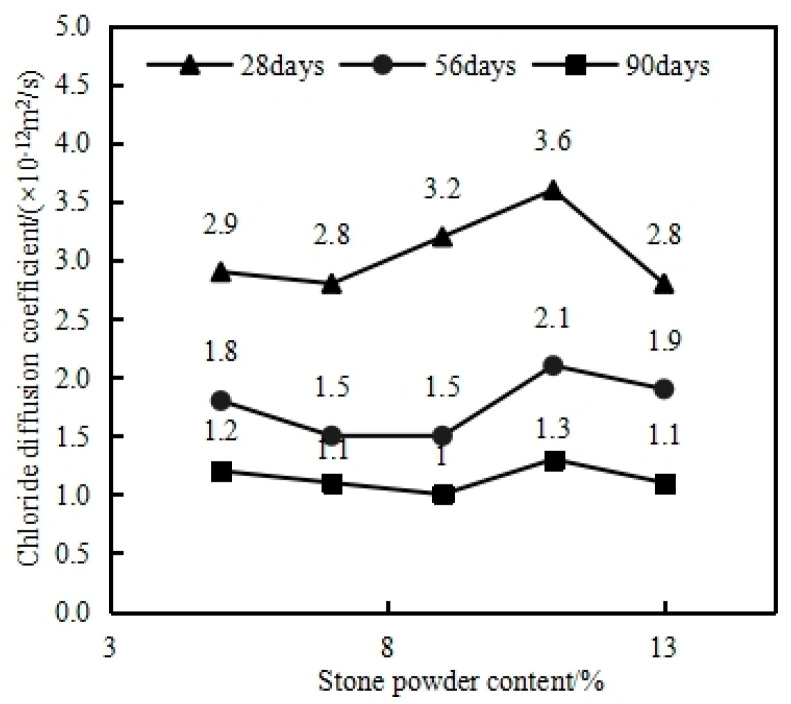
Effect of the stone powder content on the MSHPC chloride ion diffusion coefficient.

The chloride diffusion coefficient is a crucial durability parameter for the concrete structure of the immersed tube tunnel in Dalian Bay. It directly affects whether the structure can achieve the 100-year service life design requirement. Figure 14 presents the results of the chloride diffusion coefficient obtained using the Rapid Chloride Migration (RCM) method for MSHPC with varying stone powder contents at 28, 56, and 90 days. In this study, the water–binder ratio of the HPC was 0.32, with a 15% fly ash and 20% slag content. As shown in the figure, for MSHPC with a standard curing age of 28 days, the lowest chloride diffusion coefficient (2.8 × 10^−12^ m^2^/s) was observed when the stone powder content was 7%, while the highest coefficient (3.6 × 10^−12^ m^2^/s) occurred with a 11% stone powder content. By the time the curing age reached 90 days, the chloride diffusion coefficient for MSHPC with a 7–9% stone powder content decreased to 1.0–1.1 × 10^−12^ m^2^/s. In contrast, the highest chloride diffusion coefficient at 90 days was 1.3 × 10^−12^ m^2^/s, corresponding to the HPC with a 11% stone powder content. The observed outcome may be attributed to the presence of fly ash in the MSHPC, which contains a substantial quantity of active silica and alumina. These elements can undergo a secondary reaction with the byproduct of cement hydration. This reaction can result in the formation of additional gel crystals, which may enhance the pore structure of concrete. At the same time, stone powder has the function of filling microholes and improving the density of concrete. The reduction in these micropores reduces the chloride ion electric flux and the diffusion coefficient, thereby improving the anti-chloride ion penetration performance of concrete [28].

#### 4.7.2. Effect of Water–Binder Ratio on Chloride Diffusion Coefficient of MSHPC

**Figure 15:**
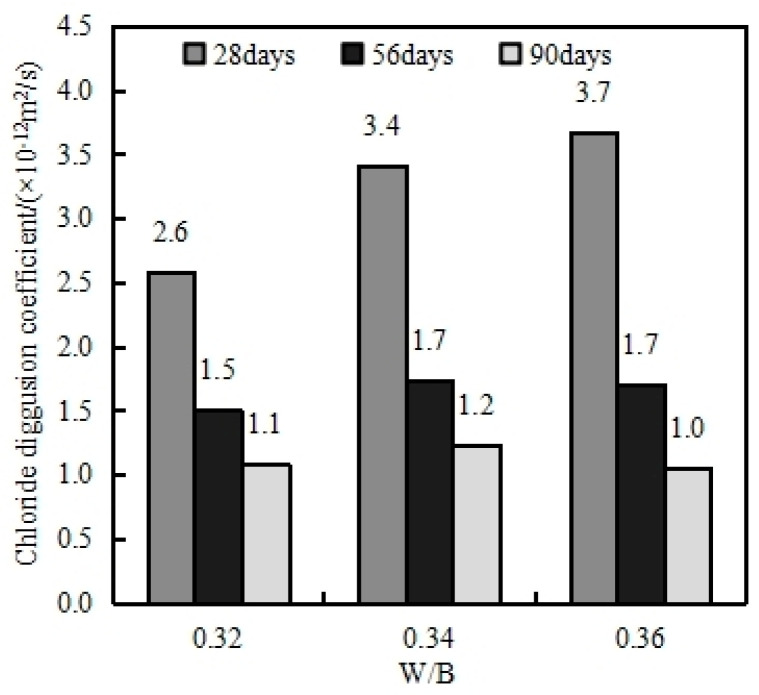
Effect of W/B on MSHPC chloride diffusion coefficient.

Figure 15 illustrates the influence of the water–binder ratio on the chloride diffusion coefficient of MSHPC, determined using the RCM method. In this study, the HPC mixtures contained 15% fly ash, 15% slag, and 9% stone powder. The results indicate that a lower water–binder ratio corresponds to a reduced chloride diffusion coefficient in MSHPC. For HPC with a water–binder ratio of 0.32, the chloride diffusion coefficients at 28, 56, and 90 days were 2.6 × 10^−12^ m^2^/s, 1.5 × 10^−12^ m^2^/s, and 1.1 × 10^−12^ m^2^/s, respectively. According to the GB/T 50476-2019 Code for Design of Concrete Structural Durability, for concrete in regions like Dalian, which are exposed to marine tidal zones and wave splash zones, the 28-day chloride ion diffusion coefficient should meet the following technical requirement: D28 ≤ 4.0 × 10^−12^ m^2^/s; this is to ensure a 100-year service life.

Based on the experimental results, the RCM chloride diffusion coefficients of MSHPC with water–binder ratios ranging from 0.32 to 0.36 and a stone powder content ranging between 9% and 13% comply with the design specifications for durability in marine environments [29].

### 4.8. Application of MSHPC in the Dalian Bay Submarine Immersed Tube Tunnel

**Figure 16:**
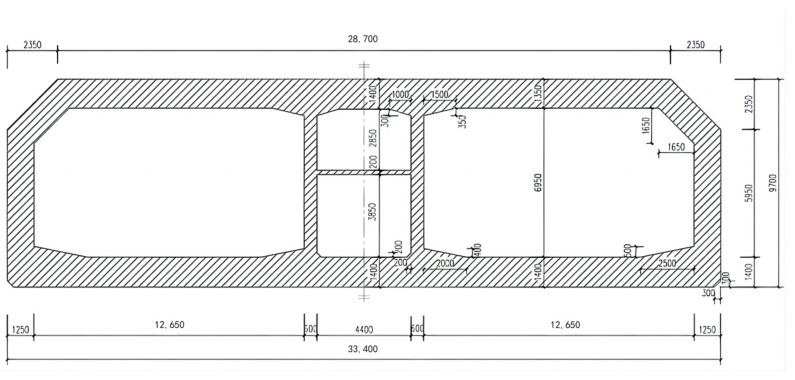
Cross section of immersed pipe structure [18].

The Dalian Bay submarine tunnel project is the first of its kind in the Dalian region. It starts at Barracuda Bay Road 20 in the north and terminates at People’s Road in the south. The total length of the tunnel is 4857 m, with a 3035 m undersea immersed tube section. The submarine immersed tube is made of a prefabricated, reinforced HPC structure using mechanized sand. The tube section is designed with a two-hole, one-tube gallery configuration, with external dimensions of 33.4 m by 9.7 m, as illustrated in Figure 16. The structure is designed to have a service life of 100 years.

**Figure 17:**
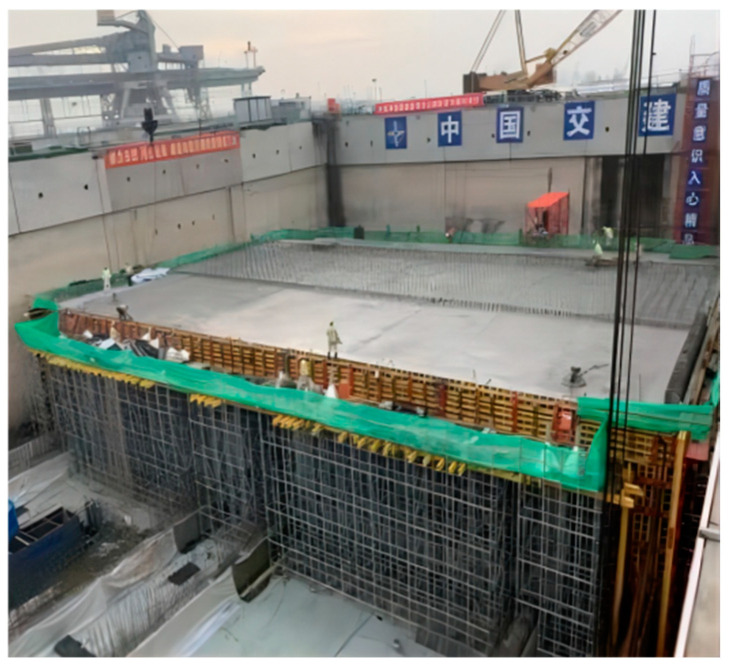
Construction drawing of the Dalian Bay submarine immersed tube tunnel project.

The Dalian Bay Cross-Harbor Tunnel’s immersed tube concrete structure is an exceptionally long and large structure, which requires a significant amount of concrete and a long pouring duration. Construction drawing of the Dalian Bay submarine immersed tube tunnel project is shown in Figure 17. This imposes strict requirements on the concrete’s workability, including its consistency and setting time. To meet these demands, the mix design of MSHPC was further optimized in the immersed tube prefabrication yard, based on an analysis of the proportioning tests and on-site casting model validation data.

A slow-setting water-reducing agent was used to control the setting time of the MSHPC, ensuring it fell within the 16–20 h range, which is crucial for smooth construction. Through experimental studies on the workability, early crack resistance, strength, and durability of HPC with different mix ratios, the following basic mix proportions for the MSHPC used in the concrete structure of the submarine immersed tube tunnel in Dalian Bay were determined: water–cementitious material ratio of 0.34, mineral admixture content of ≤40%, and mechanized sandstone powder content of ≤10%. To ensure the constructability of the HPC immersed tube, the initial mix ratio was optimized to prolong the initial setting time and demolding time, and also enhence the impermeability of concrete. By adjusting the dosage of the slow-setting water-reducing agent, the initial setting time was controlled at ≥14 h, the demolding time at ≥6 h, and the water penetration rate at ≤1%.

The preliminary factory mix scheme for the precast HPC with mechanized sand is presented in Table 6, while the optimized mix scheme is shown in Table 7. The basic physico-mechanical properties of the optimized mix are summarized in Table 8. The design strength grade reaches up to C50.

The Dalian Bay submarine tunnel was constructed using 18 large-scale immersed tube sections. These sections were precast using the finalized optimized mix ratio of mechanized sand HPC. During construction, it was important to maintain an air content of 5.0 ± 0.5% within the mold, allow a demolding time of at least 6 h, achieve an initial setting time between 16 to 20 h, maintain a slump of 220 mm ± 10 mm, and achieve a zero bleeding rate. Air content is a crucial factor in ensuring the concrete’s resistance to freezing and salt freezing.

**Figure 18:**
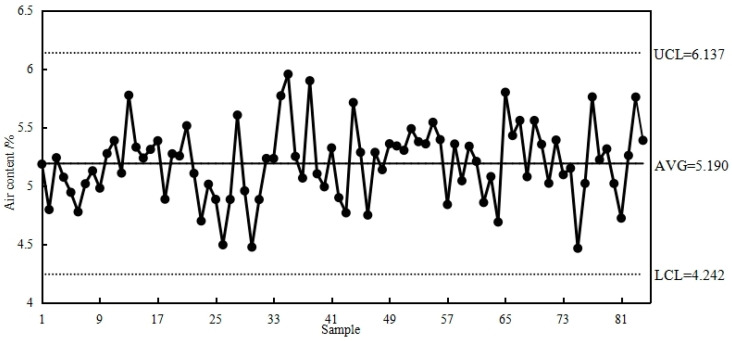
Control chart for air content during the molding of MSHPC [26].

Figure 18 shows the continuous control chart of the air content in the mechanized sand HPC during the immersed tube prefabrication process [30]. The data indicate that the average air content during casting was 5.19%, with a standard deviation of 0.316%. The air content range, controlled within three standard deviations, was between 4.242% and 6.137%, demonstrating that the frost resistance of the precast immersed tube HPC meets the design requirements.

During the on-site testing of large-scale MSHPC in immersed tube structures, the following mechanical properties and durability were observed: the 28-day and 56-day compressive strengths reached C45 and C50, respectively; the 56-day impermeability grade was P14; the 56-day electrical flux was 580C; the 56-day chloride ion diffusion coefficient was 1.9 × 10^−12^ m^2^/s; and the frost durability index was 92%. During the prefabrication of the immersed tube, the continuous monitoring of 40 batches of mechanized sand HPC with an air content ranging from 4% to 6% revealed an average 28-day compressive strength of 61.7 MPa, with a standard deviation of 3.2 MPa. The compressive strength with a 95% confidence level was 56.4 MPa, indicating a stable strength grade of C50. The concrete structure of the immersed tunnel was designed with a protective layer thickness of 75 mm. Analysis and calculation using ChaDuraLife V1.0 software [31] indicated that the structure meets the design requirements for a service life of 100 to 120 years in the wave splash and tidal zones of the Dalian Bay marine environment.

## 5. Conclusions

(1) The stone powder content in MSHPC significantly influences its physical and mechanical properties, as well as its workability. Optimal workability is achieved when the stone powder content is approximately 9%, leading to the highest slump. However, increasing the stone powder content has a negative impact on early crack resistance. An appropriate amount of stone powder can enhance the compressive strength of mechanized sand HPC but does not significantly affect the splitting tensile strength. The compressive strength of mechanized sand HPC can achieve a grade of C50 within the stone powder content range of 5% to 13%. The highest compressive strength (up to 60 MPa) for concrete with a 28-day curing period is observed at a 13% stone powder content.

(2) The stone powder content has a minimal impact on the impermeability and freeze resistance of mechanized sand HPC. The lowest chloride ion diffusion coefficient, as measured by the Rapid Chloride Migration (RCM) method, is observed when the stone powder content is between 7% and 9%. Additionally, a lower water–binder ratio enhances the concrete’s resistance to chloride ion diffusion and permeability.

(3) The concrete structure of the Dalian Bay submarine immersed tube tunnel was constructed using an HPC mix ratio of mechanized sand, determined through systematic laboratory research and on-site optimization. The finalized mix design parameters are as follows: a water–binder ratio of 0.34, a stone powder content of 9%, and a total cementitious material content of 419 kg/m^3^, including 293 kg/m^3^ of cement and 63 kg/m^3^ each of fly ash and ground slag. The mix also includes 754 kg/m^3^ of mechanized sand and 1123 kg/m^3^ of crushed stone. During the large-scale immersed tube precast construction process, the key control parameters included an air content of 5.0 ± 0.5%, a demolding time of ≥6 h, an initial setting time of 16–20 h, a slump of 220 mm ± 10 mm, and a bleeding rate of 0%.

Based on on-site engineering tests, the 28-day and 56-day compressive strengths of the immersed tube and mechanized sand concrete structure in the Dalian Bay tunnel reached C45 and C50, respectively. Additionally, the 56-day impermeability grade was P14, the 56-day electric flux was 580 C, the 56-day chloride diffusion coefficient was 1.9 × 10^−12^ m^2^/s, and the frost durability index was 92%, meeting the design requirements for a 100-year service life.

## Figures and Tables

**Table 1:** Basic physical properties of cement.

Cement	Compare Surface Area/(m^2^·kg^−1^)	Water Demand/%	Setting Time /min		Flexural Strength /MP_a_		Compressive Strength /MP_a_	
			Initial Set	Final Set	3 d	28 d	3 d	28 d
P·O	347	27.9	180	245	5.9	8.6	30.9	53.1

**Table 2:** Chemical composition of the major cementitious materials (mass percentage/%).

Cementing Material	SiO_2_	Al_2_O_3_	CaO	MgO	SO_3_	Fe_2_O_3_	Na_2_O	MnO	TiO_2_	K_2_O	I.L
P·O	31.44	5.22	49.82	2.10	2.50	4.39	0.30	0.20	0.04	0.20	3.50
FA	58.30	16.87	3.12	1.37	1.00	7.16	0.79	0.06	1.29	0.52	0.60
SG	42.66	7.86	34.26	1.86	0.82	2.71	0.47	3.71	0.02	0.12	1.22

**Table 3:** Mix ratio and mix properties of mechanical sand HPC.

NO.	W/B	Stone Powder (%)	Amount of Raw Materials for Concrete Mix of Unit Volume/kg·m^−3^								Slumps (mm)	Air Content (%)
			Cement	Water	Manufactured Sand	Stone	Fly Ash	Slag	Water Reducer	Air Entraining Agent		
JA5-1	0.32	5	294	134.5	744	1151	63 (15%)	63 (15%)	11.34	1.71	210	5.0
JA7-1	0.32	7	294	134.5	744	1151	63 (15%)	63 (15%)	11.34	1.71	225	4.8
JA9-1	0.32	9	294	134.5	744	1151	63 (15%)	63 (15%)	12.18	1.84	230	5.4
JA11-1	0.32	11	294	134.5	744	1151	63 (15%)	63 (15%)	12.18	1.84	215	4.5
JA13-1	0.32	13	294	134.5	744	1151	63 (15%)	63 (15%)	12.18	1.84	215	4.5
JA5-2	0.32	5	273	134.5	744	1151	63 (15%)	84 (20%)	11.34	1.71	220	4.7
JA7-2	0.32	7	273	134.5	744	1151	63 (15%)	84 (20%)	11.34	1.71	220	4.7
JA9-2	0.32	9	273	134.5	744	1151	63 (15%)	84 (20%)	12.18	1.84	220	4.8
JA11-2	0.32	11	273	134.5	744	1151	63 (15%)	84 (20%)	12.18	1.84	215	4.6
JA13-2	0.32	13	273	134.5	744	1151	63 (15%)	84 (20%)	12.18	1.84	215	4.5
JB9-1	0.34	9	292	142	737	1139	63 (15%)	63 (15%)	9.61	1.7	225	4.5
JC9-1	0.36	9	293	151	727	1124	63 (15%)	63 (15%)	8.38	1.44	220	5.0

**Table 4:** Chemical composition of Dalian seawater (mg/L).

PH	Cl^−^	SO_4_^2−^	Na^+^	K^+^	CO_3_^2−^	HCO_3_^−^	Ca^2+^	Mg^2+^	Undissolved Substance	Soluble Total Solid
7.8	19,179.4	2481.1	10,633.7	384.8	17.7	99.6	413	1612	688	35,137

**Table 5:** Results for the early induction of MSHPC (W/B = 0.32).

NO.	Stone Powder (%)	Crack Counts per Unit Area	Average Crack Area per Crack (mm^2^)	Total Crack Area per Unit Area (mm^2^/m^2^)	Anti-Cracking Grade
JA5-1	5	18	4.46	80.26	L-V
JA7-1	7	15	9.16	137.43	L-IV
JA9-1	9	18	10.29	185.26	L-IV
JA11-1	11	15	21.72	325.75	L-IV
JA13-1	13	15	24.50	367.52	L-IV

**Table 6:** Preliminary mix ratio of the MSHPC immersed tube in the Dalian Bay undersea tunnel project.

NO.	Gel Material (kg/m^3^)	W/B	Cement (%)	Fly Ash (%)	Slag (%)
1#	438	0.34	65	20	15
2#	420	0.34	70	15	15
3#	420	0.33	55	15	30

**Table 7:** Optimized mix ratio of MSHPC in the Dalian Bay undersea tunnel project (kg/m^3^).

Gel Material	Cement	Water	Fly Ash	Slag	Sand	Stone (5–10 mm)	Stone (10–20 mm)	Water Reducer	Air Entraining Agent
419	293	142	63	63	754	225	898	8.38	1.44

**Table 8:** Mechanical and working properties of the MSHPC with an optimized mix ratio.

Mix Ratio Number	Compressive Strength/MPa				Appearance Density/kg·m^3^	Slumps/mm	Air Content/%
	3 d	7 d	28 d	56 d			
JB9-1	28.7	41.7	58.2	62.5	2390	210	5.3

## Data Availability

The original contributions presented in the study are included in the article, further inquiries can be directed to the corresponding author.
